# Transovarial Transmission of Heartland Virus by Invasive Asian Longhorned Ticks under Laboratory Conditions

**DOI:** 10.3201/eid2803.210973

**Published:** 2022-03

**Authors:** Wilson R. Raney, Josiah B. Perry, Meghan E. Hermance

**Affiliations:** University of South Alabama College of Medicine, Mobile, Alabama, USA

**Keywords:** Heartland virus, Dabie bandavirus, Haemaphysalis longicornis, ticks, invasive species, severe fever with thrombocytopenia syndrome virus, tick-borne diseases, vector-borne infections, zoonoses

## Abstract

We demonstrated experimental acquisition and transmission of Heartland bandavirus by *Haemaphysalis longicornis* ticks. Virus was detected in tick salivary gland and midgut tissues. A total of 80% of mice exposed to 1 infected tick seroconverted, suggesting horizontal transmission. *H. longicornis* ticks can transmit the virus in the transovarial mode.

The Asian longhorned tick, *Haemaphysalis longicornis*, is an ixodid tick native to Southeast Asia that was reported in the United States during 2017 and has since been found in 17 states (*1*,*2*). In its native range, this tick is the main vector of Dabie bandavirus (*3*) (formerly severe fever with thrombocytopenia syndrome virus), the agent that causes severe human illnesses characterized by high fever, thrombocytopenia, leukopenia, and multiorgan dysfunction (*4*).

Dabie bandavirus is closely related genetically to Heartland bandavirus (HRTV) (*5*), an emerging North American virus reported during 2012 after 2 men in Missouri, USA, showed febrile illness with fatigue, thrombocytopenia, and leukopenia after exposure to ticks (*6*). Because the current geographic range and the predicted range expansion of invasive *H. longicornis* ticks overlap considerably with human cases of HRTV, including Missouri (*7*,*8*), this study was designed to assess the ability of this invasive tick species to maintain and transmit HRTV.

## The Study

**Figure 1:**
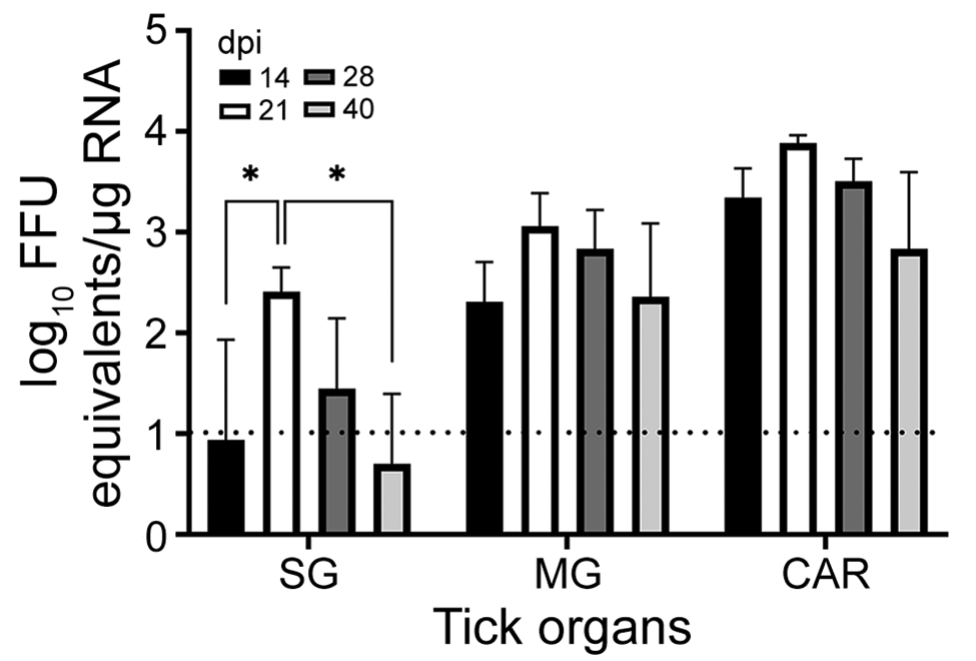
Detection of Heartland virus (HRTV) RNA by real-time, quantitative reverse transcription PCR reaction of HRTV-injected *Haemaphysalis longicornis* ticks. Ticks were dissected at 14, 21, 28, and 40 dpi. Tick organs were screened individually. Viral load data are expressed as FFU equivalents per microgram of RNA after normalization to a standard curve. Data were not normally distributed and are presented as medians with interquartile ranges. Statistical significance was determined by using Kruskal-Wallis tests followed by the Dunn test. Limit of detection was ≈10 FFU equivalents/μg RNA. *p<0.05. CAR, carcass; dpi, days postinjection; FFU, focus-forming units; MG, midgut; SG, salivary glands.

We selected 74 female *H. longicornis* ticks from an HRTV-free colony into experimental and control groups. We microinjected 50 ticks with 300 focus-forming units of HRTV into the anal pore and 24 ticks with an equivalent volume of *Dulbecco modified Eagle medium into the anal pore (*Appendix). *We dissected t*icks at 14, 21, 28, and 40 days postinjection (dpi) and collected the salivary glands, midgut, and carcass of each tick. We screened tick samples for HRTV RNA by using quantitative reverse transcription PCR (qRT-PCR) (Table 1; Figure 1). No samples taken from media-injected ticks screened positive for HRTV (Table 1). For virus-injected ticks, HRTV RNA titers followed a general trend across each organ, and titers peaked at 21 dpi (Figure 1).

**Table 1:** Rate of detection of HRTV RNA by qRT-PCR and infectious HRTV by FFA in adult *Haemaphysalis* *longicornis* ticks at 14, 21, 28, and 40 dpi*

Procedure	Real-time qRT-PCR detection of HRTV RNA, no. positive/no. tested (%)			FFA titration of HRTV, whole tick
	Salivary glands	Midgut	Carcass	
Medium injected	0/15 (0)	0/15 (0)	0/15 (0)	0/9 (0)
HRTV-injected 14 dpi	4/8 (50)	8/8 (100)	8/8 (100)	5/5 (100)
HRTV-injected 21 dpi	7/8 (88)	8/8 (100)	8/8 (100)	5/5 (100)
HRTV-injected 28 dpi	6/8 (75)	8/8 (100)	8/8 (100)	5/5 (100)
HRTV-injected 40 dpi	3/6 (50)	6/6 (100)	6/6 (100)	4/5 (80)

*dpi, days postinjection; FFA, focus-forming assay; HRTV, Heartland virus; qRT-PCR, quantitative reverse transcription PCR.

To screen HRTV-microinjected ticks for infectious virions, we collected ticks at 14, 21, 28, and 40 dpi and individually homogenized them. We cultured tick homogenates in triplicate on Vero E6 cells, and titered infectious virus by using a focus-forming assay (FFA). All ticks from each time point produced foci, indicating the presence of infectious virions in the tick body at each interval (Table 1).

We selected an additional 26 female *H. longicornis* ticks to evaluate horizontal transmission of HRTV to BALB/c mice. We microinjected 16 ticks with HRTV and the remaining 10 with *Dulbecco modified Eagle medium*. At 40 dpi, mice were infested with the microinjected ticks at a ratio of 1 tick/mouse. Five of the HRTV-injected ticks and 5 medium-injected ticks attached and fed on the mice to repletion. After feeding was complete, we removed engorged ticks and housed them individually to aid oviposition. We monitored mice daily for clinical signs of disease. We collected blood from the mice at −1, 7, and 14 days after tick attachment. Mice were subjected to necropsy at 28 days after attachment, and we collected liver, spleen, kidney, brain, blood, and testis samples. We screened blood and organ samples for HRTV RNA by qRT-PCR. No HRTV RNA was detected in any blood or organs collected from the mice.

We screened serum from the terminal blood samples to determine whether mice seroconverted relative to HRTV. We assayed each serum sample on 2 independent occasions. In brief, we assayed diluted serum samples by using HRTV-infected Vero E6 cells as antigens. Four of the 5 mice fed upon by a single HRTV-injected tick were positive for HRTV-specific antibodies. We detected antibodies up to a serum dilution of 1:1,600 for 3 mice and 1:800 for 1 mouse. None of the 5 mice fed upon by media-injected ticks were positive for HRTV-specific antibodies. Likewise, none of the age-matched, sex-matched, preimmune mouse serum demonstrated an antibody response to HRTV.

**Figure 2:**
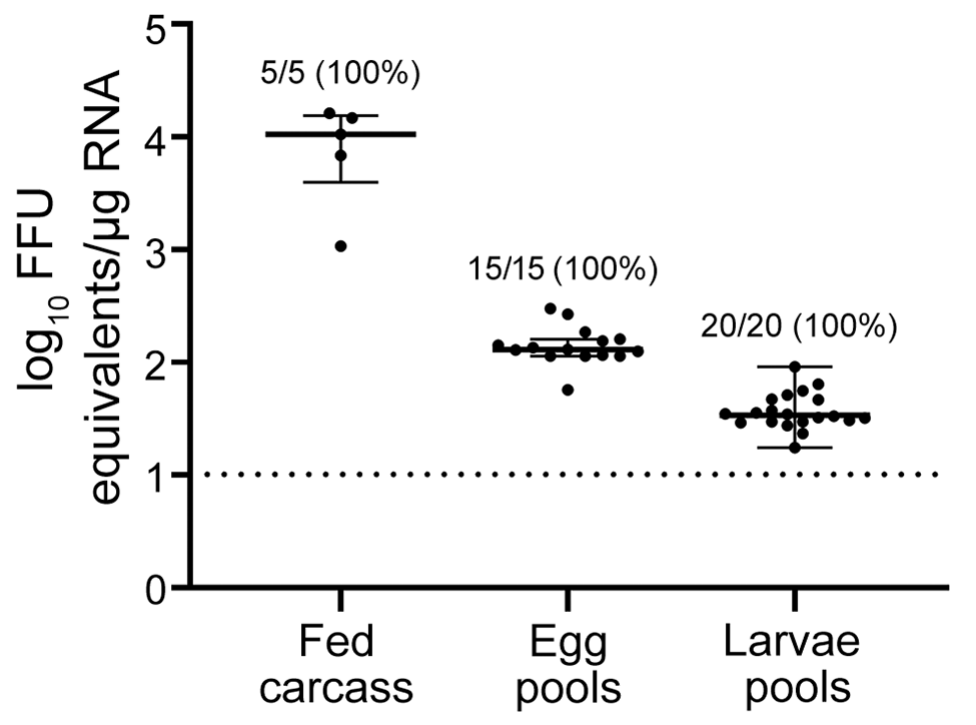
Scatter plot demonstrating detection of Heartland virus (HRTV) by real-time, quantitative reverse transcription PCR. Data were not normally distributed and are presented as medians with interquartile ranges. Fed female carcasses, egg pools, and larvae pools were screened for viral RNA. Egg pools and larvae pools were composed of 50 eggs or larvae per pool. Limit of detection was ≈10 FFU equivalents/μg RNA. FFU, focus-forming units.

After each fed female tick completed oviposition, we removed the fed female carcass from the egg mass and homogenized the carcass. We screened the carcasses for HRTV RNA by qRT-PCR, and 5/5 HRTV-injected female carcasses were positive for HRTV RNA (Table 2; Figure 2). The media-injected fed female carcasses were negative for HRTV. We also removed 3 pools of 50 eggs/egg mass to screen for HRTV RNA. All 15 egg pools from HRTV-injected ticks were positive for HRTV RNA (Table 2; Figure 2). Egg pools from the media-injected ticks had no HRTV RNA.

**Table 2:** Rate of detection of HRTV RNA by qRT-PCR and infectious HRTV by FFA in fed *Haemaphysalis* *longicornis* adult tick carcasses, tick eggs, and tick larvae*

Procedure	Real-time qRT-PCR detection of HRTV RNA, no. positive/no. tested (%)			FFA titration of HRTV, larvae pools‡
	Fed adult carcasses	Egg pools†	Larvae pools†	
Medium injected	0/5 (0)	0/15 (0)	0/20 (0)	0/5 (0)
HRTV-injected	5/5 (100)	15/15 (100)	20/20 (100)	5/5 (100)

*FFA, focus-forming assay; HRTV, Heartland virus; qRT-PCR, quantitative reverse transcription PCR. † Egg and larvae pools contained 50 eggs or larvae/pool. ‡Larvae pools contained 150 larvae/pool.

We repeated this analysis for pools of larvae (4 pools of 50 larvae derived from each fed female) after hatching. All larvae clutches derived from HRTV-injected females were positive for HRTV RNA. To screen for infectious virions in larvae, we homogenized pools of 150 larvae from each clutch and cultured them on Vero E6 cells. We titered the infectious virus by using FFA, and all 5 clutches derived from HRTV-injected females were positive for infectious HRTV (Table 2).

## Conclusions

We demonstrated experimental acquisition and transmission of HRTV by *H. longicornis* ticks after microinjection of the anal pore with HRTV. Although not a natural route of virus acquisition for ticks, microinjection of the anal pore is an established and reproducible procedure that delivers specific quantities of virus into the alimentary canal of the tick, the first organ system that virus contacts in naturally infected ticks (*9*). Microinjected ticks showed viral RNA titers peaking at 21 dpi in salivary glands, midguts, and carcasses, suggesting that HRTV replication took place within these organs between 14 and 21 dpi.

Maintenance of infectious HRTV virions for several weeks after microinjection suggests that an artificially infected tick is capable of transmitting HRTV to vertebrate hosts on which it feeds long after viral acquisition. Although the mice exposed to HRTV-infected ticks did not show clinical signs of disease and viral RNA was not detected in any mouse tissues, the absence of disease in these immunocompetent mice was expected; previously, only immunocompromised Ag129 mice have shown detectable viremia, clinical signs of HRTV infection, and death (*10*). Seroconversion of 4/5 mice exposed to an individual HRTV-infected *H. longicornis* tick suggests horizontal transmission of HRTV. Future studies should confirm the presence of infectious virions in tick saliva to eliminate the possibility of seroconversion caused by transmission of noninfectious HRTV antigens during tick feeding.

We also showed transovarial transmission of HRTV in *H. longicornis* ticks by detection of HRTV RNA in eggs and larvae derived from HRTV-infected mother ticks. Furthermore, we demonstrated the presence of infectious virions in larvae after hatching. The North American strain of the tick is parthenogenetic, a foremost public health concern because 1 female can reproduce asexually to establish and sustain local populations (*11*). Because *H. longicornis* ticks are a 3-host tick and a host generalist (*12*), the possibility of invasive *H. longicornis* ticks acquiring HRTV by cofeeding with infected ticks or by feeding on a viremic host further highlights the potential of the tick to efficiently disseminate the virus. This distinction becomes more crucial because the tick can withstand a wide range of climates (*7*,*13*). Further studies should be conducted to demonstrate whether the tick can transmit HRTV during co-feeding with other ticks because this would be a major factor in promoting the environmental spread of the virus.

The predicted spread of *H. longicornis* ticks in the United States shares a geographic range with states in which HRTV has already been reported in *Amblyomma americanum* ticks and wildlife (*7*,*8*,*14*). The introduction of a new vector species could amplify transmission in natural foci, resulting increased HRTV disease cases in humans.

Appendix Additional information on transovarial transmission of Heartland virus by invasive Asian longhorned ticks under laboratory conditions.

## References

[1] US Department of Agriculture. National *Haemaphysalis longicornis* (Asian longhorned tick) situation report as of September 10, 2021 [cited 2021 Dec 8]. https://www.aphis.usda.gov/animal_health/animal_diseases/tick/downloads/longhorned-tick-sitrep.pdf

[2] Rainey T, Occi JL, Robbins RG, Egizi A. Discovery of Haemaphysalis longicornis (Ixodida: Ixodidae) Parasitizing a Sheep in New Jersey, United States. J Med Entomol. 2018;55:757–9. 10.1093/jme/tjy006 29471482

[3] Liu K, Zhou H, Sun RX, Yao HW, Li Y, Wang LP, et al. A national assessment of the epidemiology of severe fever with thrombocytopenia syndrome, China. Sci Rep. 2015;5:9679. 10.1038/srep09679 25902910 PMC4407178

[4] Yu XJ, Liang MF, Zhang SY, Liu Y, Li JD, Sun YL, et al. Fever with thrombocytopenia associated with a novel bunyavirus in China. N Engl J Med. 2011;364:1523–32. 10.1056/NEJMoa1010095 21410387 PMC3113718

[5] Matsuno K, Weisend C, Travassos da Rosa AP, Anzick SL, Dahlstrom E, Porcella SF, et al. Characterization of the Bhanja serogroup viruses (Bunyaviridae): a novel species of the genus Phlebovirus and its relationship with other emerging tick-borne phleboviruses. J Virol. 2013;87:3719–28. 10.1128/JVI.02845-12 23325688 PMC3624231

[6] McMullan LK, Folk SM, Kelly AJ, MacNeil A, Goldsmith CS, Metcalfe MG, et al. A new phlebovirus associated with severe febrile illness in Missouri. N Engl J Med. 2012;367:834–41. 10.1056/NEJMoa1203378 22931317

[7] Raghavan RK, Barker SC, Cobos ME, Barker D, Teo EJM, Foley DH, et al. Potential spatial distribution of the newly introduced long-horned tick, *Haemaphysalis longicornis*, in North America. Sci Rep. 2019;9:498. 10.1038/s41598-018-37205-2 30679711 PMC6346113

[8] Staples JE, Pastula DM, Panella AJ, Rabe IB, Kosoy OI, Walker WL, et al. Investigation of Heartland virus throughout the United States, 2013‒2017. Open Forum Infect Dis. 2020;7:ofaa125. 10.1093/ofid/ofaa125 PMC7246346 32478118

[9] Labuda M, Nuttall PA. Tick-borne viruses. Parasitology. 2004;129(Suppl):S221–45. 10.1017/S0031182004005220 15938513

[10] Bosco-Lauth AM, Calvert AE, Root JJ, Gidlewski T, Bird BH, Bowen RA, et al. Vertebrate host susceptibility to Heartland virus. Emerg Infect Dis. 2016;22:2070–7. 10.3201/eid2212.160472 27869591 PMC5189141

[11] Egizi A, Bulaga-Seraphin L, Alt E, Bajwa WI, Bernick J, Bickerton M, et al. First glimpse into the origin and spread of the Asian longhorned tick, *Haemaphysalis longicornis*, in the United States. Zoonoses Public Health. 2020;67:637–50. 10.1111/zph.12743 32638553

[12] Hoogstraal H, Roberts FH, Kohls GM, Tipton VJ. Review of *Haemaphysalis* (*kaiseriana*) *Longicornis Neumann* (resurrected) of Australia, New Zealand, New Caledonia, Fiji, Japan, Korea, and Northeastern China and USSR, and its parthenogenetic and bisexual populations (Ixodoidea, Ixodidae). J Parasitol. 1968;54:1197–213. 10.2307/3276992 5757695

[13] Heath A. Biology, ecology and distribution of the tick, *Haemaphysalis longicornis Neumann* (Acari: Ixodidae) in New Zealand. N Z Vet J. 2016;64:10–20. 10.1080/00480169.2015.1035769 25849758

[14] Brault AC, Savage HM, Duggal NK, Eisen RJ, Staples JE. Heartland virus epidemiology, vector association, and disease potential. Viruses. 2018;10:E498. 10.3390/v10090498 30223439 PMC6164824

