# Supplementary material for: Transovarial Transmission of Heartland Virus by Invasive Asian Longhorned Ticks under Laboratory Conditions
Source: Emerg Infect Dis. 2022 Mar;28(3):726–9. doi: 10.3201/eid2803.210973 (PMC8888223; doi:10.3201/eid2803.210973)
Supplement: Appendix — Additional information on transovarial transmission of Heartland virus by invasive Asian longhorned ticks under laboratory conditions. [file 21-0973-Techapp-s1.pdf]

Article DOI: https://doi.org/10.3201/eid2803.210973

Transovarial Transmission of Heartland Virus by Invasive Asian Longhorned Ticks under Laboratory Conditions

Appendix

Materials and Methods

Ethics

All studies including mice were conducted in animal biosafety level 3 (ABSL-3) facilities. Bloodmeals were provided to uninfected ticks for colony maintenance by feeding on rabbits in animal biosafety level 2 (ABSL-2) facilities. Work involving mice and rabbits was conducted with animal use protocols approved by the University of South Alabama Institutional Animal Care and Use Committee (nos. 1619216–2 and 1619233–2)

Cells and Virus

A culture of African green monkey kidney (Vero E6) cells was maintained in Dulbecco modified Eagle medium (DMEM) supplemented with 10% fetal bovine serum and 1% penicillin/streptomycin. Cell culture was incubated at 37°C in an atmosphere of 5% CO_2. Heartland virus (HRTV) strain MO-4 was acquired from the World Reference Center for Emerging Viruses and Arboviruses at the University of Texas Medical Branch (Galveston, TX, USA). The stock had previously been passaged once on DH82 cells, once on Vero cells, and once in suckling mouse brain. The stock was then passaged on Vero E6 cells 3 times. Focus-forming assays (FFAs) were used to determine stock virus titers as described (*1*).

Ticks and Animals

Adult female *Haemaphysalis longicornis* ticks (BEI Resources, https://www.beiresources.org) were maintained by feeding on New Zealand White rabbits (Charles River Laboratories, https://www.criver.com). Uninfected ticks were housed under arthropod containment level 2 (ACL-2) conditions. Infected ticks were housed under ACL-3 conditions in a negative pressure containment enclosure (bioBUBBLE Inc.,

https://biobubble.com). ACL-2 and ACL-3 facilities were maintained at 23°C and on a 16:8 light:dark cycle. Tick vials were housed in a high-humidity desiccator at $\geq$90% relative humidity.

Male and female BALB/c mice were obtained from The Jackson Laboratory (https://www.jax.org). Mice acclimated to the environment for a minimum of 5 days before experimentation. Mice were 10 weeks old upon the start of tick infestation and ranged from 10 to 11 weeks old at the time of tick attachment and initiation of tick feeding. Mice were weighed before and after capsule attachment and were randomly assigned to each infection group. After tick capsules were attached to mice, each mouse was individually housed in ventilated cage systems. A 12:12 light:dark cycle was maintained for all cages. Temperature and humidity were closely regulated for the cage system. Food and water were provided to each mouse ad libitum.

Tick Viral Replication Kinetics

Infection of Adult Ticks by Anal Pore Microinjection

Adult female *H. longicornis* ticks were microinjected in the anal pore with HRTV or medium as described (*2*). In brief, infection by microinjection was accomplished by injecting 475 nL of virus stock containing ≈300 FFUs of HRTV into the anal aperture of the immobilized tick by using a digitally-controlled microinjector with a footswitch, glass microneedles, and a dissecting microscope. An equivalent volume of DMEM was used to microinject ticks in the mock-infected control group. All microinjected ticks were the same generation and same time post-molt (i.e., 6 weeks post-molting from nymph to adult). After microinjection, all ticks were maintained in the ACL-3. Tick viability was monitored twice daily for 4 days after microinjection.

Tick Dissection

At 14, 21, 28, and 40 days postinjection, ticks were dissected from the virus-injected and the media-injected cohorts. Salivary glands and midgut were collected from each tick, and the remaining tick organs, including the exoskeleton, were retained and designated as carcass.

RNA Extraction from Tick Tissues

Tick tissues (salivary glands and midguts) were homogenized in 100 µL TRIzol reagent by using a pellet pestle mixer (Thermo Fisher Scientific, https://www.thermofisher.com). Tick carcasses were homogenized in TRIzol reagent with sterile metal beads in a TissueLyser II (QIAGEN, https://www.qiagen.com) at 30 hz for 3 minutes. A hybrid of TRIzol and QIAGEN

RNeasy Mini Kit protocols was used for tissue RNA extractions because it has been demonstrated that these combined protocols inactivate virus and yield high-quality RNA (*3*). In brief, chloroform was added to tissue homogenate at 0.2 mL chloroform/1 mL homogenate. The samples were shaken vigorously for 15 seconds and incubated at room temperature for 3 minutes. Samples were centrifuged at 12,000 × *g* at 4°C for 15 minutes. The aqueous phase was removed and was mixed with 1 volume of 70% ethanol by pipetting. The samples were added to an Rneasy Mini Column (QIAGEN), and the protocol for the kit was followed. Total RNA was eluted from the column with 30 µL of nuclease-free water. RNA quantity and purity were determined by using a NanoDrop 1000 Spectrophotometer (Thermo Fisher Scientific).

Detection of HRTV RNA in Tick Tissues by Real-Time Quantitative Reverse Transcription PCR

Absolute quantification of viral loads in tick tissue samples were determined by using a real-time quantitative reverse transcription PCR (qRT-PCR) as described (*4*). In brief, viral RNA loads are expressed on a log10 scale as FFU equivalents per microgram of RNA after normalization to a standard curve produced by using serial 10-fold dilutions of viral RNA from known quantities of infectious virus to estimate viral burden. The qRT-PCR was performed by using forward (5′-CCTTTGGTCCACATTGATTG-3′) and reverse (5′-CACTGATTCCACAGGCAGAT-3′) primers specific to the HRTV spike (S) gene (*5*). An HRTV S gene probe (5′-56-FAM/TGGATGCCTATTCCCTTTGGCAA/36-TAMSp-3′) was also used. When performing a qRT-PCR, a standard amount of sample RNA was added to the appropriate wells of a 96-well PCR plate. Reagents from the iTaq Universal SYBR Green One-Step Probes Kit (BioRad Laboratories, https://www.bio-rad.com), 10 µmol/L of forward and reverse primers, and probe specific to the HRTV S gene were added to the wells. The total reaction volume per well was 20 µL. Plates were sealed and run on a QuantStudio 5 Real-Time PCR System (Thermo Fisher Scientific) at the following cycle settings: 10 minutes at 50°C; 10 seconds at 95°C; and 30 seconds for 45 cycles at 60°C.

Infectious HRTV Whole Tick FFA

Whole adult ticks were harvested at 14, 21, 28, and 40 dpi and frozen at −80°C. One pool of 150 larvae hatched from each fed female was harvested ≈2–3 weeks post-hatching and processed by using the FFA. For FFA of tick viral loads, whole ticks were individually homogenized in DMEM supplemented with 2% fetal bovine serum, 1% penicillin/streptomycin,

and 1% fungizone by using sterile metal beads in a Bead Ruptor 96 Tissue Lyser (OMNI International, https://us.omni-inc.com). Larval pools were also individually homogenized as described above. Tick homogenate was centrifuged at 5,000 × *g* at room temperature for 5 minutes. The clarified tick homogenate was then aliquoted into new sterile tubes, and 65 µL of undiluted tick homogenate was used to infect Vero E6 cells seeded in 48-well plates. For adult whole ticks, plates were infected in triplicate. For larval pools, plates were infected in duplicate. Viral titers in adult and larval *H. longicornis* tick samples were determined by using FFA as described (*2*).

HRTV Transmission from Tick to Mouse

Tick infestations on mice were performed by using capsules made of 2-ml cryotubes. The base of each tube was cut to leave ≈3 mm of the remaining tube below the screw-cap lid. The top of the lid was cut to enable an opening when secured. The base of the capsule was attached to the upper dorsum of each mouse by using athletic tape adhered with livestock Kamar glue (Kamar Inc., https://www.enasco.com). After placing ticks inside the capsules, a piece of fine mesh fabric was placed under the capsule lid before closing to enable tick containment and air circulation. Masking tape was used to secure each capsule lid. Capsule integrity was checked daily throughout the tick infestations, and capsules were reinforced with additional adhesive and bandages as needed.

Bodyweights and clinical observations were documented daily for each mouse after addition of 1 tick to each capsule. Ticks that did not attach to mice were removed from the capsule after 1 week. Engorged ticks were removed from capsules upon completion of feeding and detachment from the skin. Blood samples were collected from each mouse by submandibular bleed at −1, 7, and 14 days postattachment under isoflurane anesthesia. Blood was also collected during necropsy by terminal cardiac bleed. Changes in appearance, respiration, neurologic, and behavior were documented daily. When mice reached the study endpoint (28 days postattachment), euthanasia was performed by isoflurane overdose, followed by cervical dislocation and terminal cardiac bleed. Tissues harvested during necropsy were fixed at room temperature in 10% neutral-buffered formalin for 72 hours. Formalin was replaced after 24 hours. Necropsies of mock-infected and HRTV-infected mice were conducted under ABSL-3 conditions.

The following tissues were harvested from mice after undergoing necropsy and were stored in TRIzol Reagent (Invitrogen Life Technologies, https://www.thermofisher.com): spleen, liver, kidney, testes, and brain. Blood collected during submandibular bleeds was also stored in TRIzol Reagent. Terminal mouse blood was divided between TRIzol Reagent and serum separation tubes for storage. Mouse tissues were homogenized in a bead beater system as described above. Homogenized mouse tissues and blood were screened for viral load by qRT-PCR as described above.

An in-house immunoassay was performed to detect HRTV antibodies in serum samples from mice fed upon by HRTV-infected ticks. Vero E6 cells were infected with HRTV at a multiplicity of infection of 0.1 in 48-well plates. After a 1-hour incubation, the virus inoculum was removed, and the cells were incubated for 2 days in 2% fetal bovine serum in DMEM. The plates were fixed with 1:1 methanol:acetone for 30 minutes and air dried. The plates were washed 3 times with phosphate-buffered saline, 0.1% Tween 20 (PBST) and blocked with a 5% goat serum solution. Mouse serum was serially diluted 2-fold from 1:25 to 1:25,600. A total of 65 μL mouse serum dilution was added to the designated plate well and incubated for 1 hour. The plates were washed in PBST and then stained with a secondary goat anti-mouse horseradish peroxidase–conjugated antibody for 1 hour.

Plates were washed with PBST after secondary antibody staining. To develop the plates, 65 μL of aminoethyl carbazole developing solution (ImmPACT AEC Kit, Vector Laboratories, https://vectorlabs.com) was applied to each well according to the product instructions. Plates were wrapped in foil and developed for 30 minutes. The plates were submerged in water to stop development. Presence of HRTV antibodies were confirmed by identifying the lowest serum dilution for which a signal was observed. Comparative immunoassays were performed by using naïve preimmune serum from age-matched sex-matched mice following the same protocol. Every immunoassay included a HRTV-specific antibody positive control (in the form of mouse hyperimmune ascitic fluid) to confirm that the Vero E6 cells were infected and displayed HRTV antigen. To confirm that the secondary antibody did not bind nonspecifically to cellular components, secondary antibody only (i.e., no primary antibody) controls were included for each immunoassay. In addition, every immunoassay included mock-infected Vero E6 cells that were exposed to HRTV mouse hyperimmune ascitic fluid positive control antibody.

HRTV Transovarial Transmission

Engorged ticks were individually housed after feeding on mice. At 14 days (±1 day) post-oviposition, 3 pools of 50 eggs were collected from each egg mass. After the remaining eggs of each clutch had hatched, 4 pools of 50 larvae were collected from each clutch. Each egg and larvae pool was homogenized, and RNA was extracted from the homogenates as described above. Egg and larvae pools were screened for viral loads by using qRT-PCR as described above. For titration of infectious HRTV in larvae by FFA, pools of 150 larvae were homogenized, clarified, and cultured on Vero E6 cells as described above.

References

1. Rossi SL, Nasar F, Cardosa J, Mayer SV, Tesh RB, Hanley KA, et al. Genetic and phenotypic characterization of sylvatic dengue virus type 4 strains. Virology. 2012;423:58–67. PubMed https://doi.org/10.1016/j.virol.2011.11.018

2. Talactac MR, Yoshii K, Hernandez EP, Kusakisako K, Galay RL, Fujisaki K, et al. Synchronous Langat virus infection of *Haemaphysalis longicornis* using anal pore microinjection. Viruses. 2017;9:E189. PubMed https://doi.org/10.3390/v9070189

3. Hermance ME, Thangamani S. Tick saliva enhances Powassan virus transmission to the host, influencing its dissemination and the course of disease. J Virol. 2015;89:7852–60. PubMed https://doi.org/10.1128/JVI.01056-15

4. Hermance ME, Widen SG, Wood TG, Thangamani S. *Ixodes scapularis* salivary gland microRNAs are differentially expressed during Powassan virus transmission. Sci Rep. 2019;9:13110. PubMed https://doi.org/10.1038/s41598-019-49572-5

5. Savage HM, Godsey MS, Lambert A, Panella NA, Burkhalter KL, Harmon JR, et al. First detection of Heartland virus (Bunyaviridae: Phlebovirus) from field collected arthropods. Am J Trop Med Hyg. 2013;89:445–52. PubMed https://doi.org/10.4269/ajtmh.13-0209
